# How Learners’ Corrective Feedback Beliefs Modulate Their Oral Accuracy: A Comparative Study on High- and Low-Accuracy Learners of Chinese as a Second Language

**DOI:** 10.3389/fpsyg.2022.869468

**Published:** 2022-05-11

**Authors:** Jingwei Zhang, Xianwen Cao, Nan Zheng

**Affiliations:** ^1^Department of Chinese Language and Literature, Faculty of Arts and Humanities, University of Macau, Macao, Macao SAR, China; ^2^Institute for International Students, Nanjing University, Nanjing, China

**Keywords:** second language acquisition (SLA), Chinese as a second language (CSL), corrective feedback, oral communication, language pedagogy

## Abstract

This paper explores the differences in high-accuracy and low-accuracy learners’ beliefs about corrective feedback when learning Chinese as a second language (henceforth, CSL). In this study, we collected data through a questionnaire survey and an oral test with 76 CSL learners in a Chinese university. The analysis revealed that both high- and low-accuracy CSL learners shared the same beliefs in whether and how the learner errors should be corrected but differed in their beliefs about when is the best time to correct, which error should be corrected, and who the corrector should be. Specifically, the discrepancy between high- and low-accuracy groups’ beliefs about corrective feedback was found to be related to the participants’ oral accuracy. Our results confirm that learners’ CF beliefs can modulate their language accuracy. The corrective feedback beliefs held by high-accuracy groups have implications for improving low-accuracy groups’ oral accuracy. Through comparison with findings on corrective feedback beliefs of English as a foreign/second language (henceforth, EFL/ESL) learners, this study suggested that language pedagogies developed from the research of EFL/ESL learners’ CF beliefs should be able to shed light on this area and have significance for CSL learners. Implications for correcting learner errors in teaching CSL are also provided in the paper.

## Introduction

Corrective feedback (CF) refers to the response that learners receive about their linguistic errors made in their oral or written production in a second language (Sheen and Ellis, 2011). CF has been a key issue in language teaching and learning and language pedagogy for almost half a century. Whether CF can benefit the second language acquisition process was a highly controversial issue in the early stages in this field. Studies like Krashen (1982, 1985) and VanPatten (1992) opposed the role of CF in language learning. However, with the development of empirical research, increasing evidence has emerged to support that CF can assist language learning by improving learners’ accuracy (e.g., Bitchener et al., 2005; Ellis et al., 2006; Benson and DeKeyser, 2019; Hashemifardnia et al., 2019; Kim and Emeliyanova, 2021). One of the factors to determine the effectiveness of CF has been found to be the learner’s belief about CF (Ellis, 2010; Storch and Wigglesworth, 2010).

The term learner beliefs refers to the conceptions, ideas, and opinions learners have about second language learning and teaching and language itself (Kalaja et al., 2018). Many studies have shown that learners’ beliefs about CF can directly influence their participation in and uptake of CF, further mediating the effectiveness of CF (Leki, 1991; Kern, 1995; Schulz, 2001; Sheen, 2011; Han, 2017). However, little is known about the relationship between learners’ CF beliefs and their language performance – particularly in terms of oral accuracy. To fill this gap, the current study aims to further examine the relationship between learners’ CF beliefs and their second language accuracy.

Although extensive research has been carried out on CF beliefs, it has been mostly restricted to those of English as a foreign/second language (henceforth, EFL/ESL) learners (Lyster and Ranta, 1997; Panova and Lyster, 2002; Han and Hyland, 2015; Chong, 2019). Another gap is that little research was designed to investigate the CF beliefs of Chinese as a second language (CSL) learners. Over the past two decades, there has been a tremendous growth of learning and teaching of CSL both within and outside China (Gong et al., 2020a,b). According to the Ministry of Education of China, there are cumulatively almost 200 million non-Chinese people learning Chinese languages (Xinhua News Agency, 2020). In accordance with the increasing demand for Chinese learning, there is a growing need for CSL teachers who can meet the diverse needs of CSL learners. Understanding the CF beliefs of CSL learners is of special significance to explore the pedagogical implications. Therefore, the present study also aims to obtain a comprehensive overview of the CF beliefs of CSL learners.

Overall, this study attempts to examine the relationship between learners’ CF beliefs and their second language oral accuracy from a cohort of CSL learners. Three formal aspects of language – vocabulary, grammar and pronunciation – are used as the indicators of oral accuracy. As CF outcomes differ between high- and low-accuracy learners (Powell, 1987), these two groups of learners were examined.

## Literature Review

### Corrective Feedback Beliefs of Second Language Learners

In a seminal article, Hendrickson (1978) summarized five fundamental questions about CF: (1) Should learner errors be corrected? (Efficacy of CF) (2) If so, when should learner errors be corrected? (Timing of CF) (3) Which learner errors should be corrected? (Choice of Errors to Correct) (4) How should learner errors be corrected? (Choice of CF Strategy) and (5) Who should correct learner errors? (Choice of Correctors). These questions were addressed by studies like Ellis (2009b), Zhang and Rahimi (2014), and Zhu and Wang (2019). In the following, we will review the recent studies on CF beliefs of second language learners, mostly EFL/ESL learners, based on those five questions. Regarding the efficacy of CF, there is a growing consensus that oral CF contributes to second language acquisition, at least to a certain extent. Earlier in 1978, Hendrickson already argued that oral errors should be corrected. Long (1996, 2006) specifically pointed out that recast, one of the most important types of CF, benefits learners’ oral acquisition by attracting their attention to form while keeping learners focused on meaning throughout a conversational exchange. The positive effects of CF are supported both by empirical evidence (e.g., Doughty and Varela, 1998; Han, 2002; Loewen et al., 2009; Lee, 2013; Zhang and Rahimi, 2014; Zhu and Wang, 2019) and the meta-analyses of CF studies (Mackey and Goo, 2007; Li, 2010; Lyster and Saito, 2010).

Apart from the issue of whether errors should be corrected, many studies have moved on to discuss when errors should be corrected but the results are rather mixed. Some studies supported delayed correction (e.g., Walker, 1973; Harmer, 1983; Bartram and Walt, 1991) while other studies supported immediate correction (e.g., Davis, 2003; Zhang and Rahimi, 2014). Quinn (2014), Li et al. (2016), and Zhu and Wang (2019) have suggested that these mixed pictures may be caused by learners’ different understandings of “immediate” and “delayed” CF. It is therefore worthwhile to explore CSL learners’ beliefs about CF timing and also to ascertain if high- and low-accuracy learners have different beliefs.

As for the choice of errors to correct, Zhu and Wang (2019) summarized three taxonomies of errors based on previous studies: (1) linguistic taxonomy (e.g., grammatical, lexical, phonological errors); (2) focused CF (attention is directed to a few errors) vs. unfocused CF (all errors are addressed); and (3) the gravity of errors (whether an error impedes communication). Zhu and Wang (2019) find that “the gravity of errors might inherently be the only line of demarcation for error types in the learners’ belief system.” The current study therefore explored learners’ CF beliefs toward different gravity of errors, as did Zhu and Wang (2019).

Regarding the choice of CF strategy, oral corrective strategies can be divided into implicit feedback and explicit feedback (Sheen and Ellis, 2011). Implicit feedback is a kind of feedback without an overt marker of errors, e.g., recast, while explicit feedback uses an overt marker, e.g., metalinguistic comment. Previous studies about EFL/ESL students’ preference for the CF strategies also showed a mixed picture. Some studies found a preference for explicit correction (e.g., Katayama, 2007; Lee, 2013; Zhang and Rahimi, 2014) while some showed that learners favor implicit correction (e.g., Oladejo, 1993; Zhu and Wang, 2019). Zhu and Wang (2019) suggest that learners’ preference for CF strategies might reflect “their beliefs as to whether comprehensible input or learner output is more important for language acquisition.”

With regard to the CF provider, compared to peer-correction and self-correction, EFL/ESL learners generally ranked the teacher as the favorite choice of correctors. Apart from the teacher as corrector, some studies showed peer-correction is the second favored, e.g., Schulz (2001), Zhang and Rahimi (2014), Agudo (2015), and Zhu and Wang (2019). However, a few studies like Katayama (2007) and Yoshida (2008) show learner reluctance toward peer-correction because it is not authoritative enough.

Based on the previous literature, researchers have generated a substantial amount of research on CF beliefs of EFL/ESL learners. These, by analog, may serve as useful benchmark models to investigate CSL learners’ CF beliefs, though some findings for EFL/ESL learners revealed a rather mixed picture regarding timing of CF, choice of CF strategy and choice of correctors.

### Studies on Corrective Feedback Beliefs and Oral Accuracy

Second language acquisition is a process that involves three core dimensions – complexity, accuracy, and fluency (Skehan, 1989, 1996, 1998; Ellis, 2009c). Most studies regarding the efficiency of CF in improving accuracy lie in the research field of written accuracy (e.g., Krashen, 1982; Truscott, 1996, 1999; Ferris, 1999; Sheen, 2007; Ellis et al., 2008; Ellis, 2009a; Frear and Chiu, 2015; Shintani and Aubrey, 2016; Benson and DeKeyser, 2019; Karim and Nassaji, 2020) and focus on the accuracy of grammar and vocabulary. By contrast, the effects of CF in oral accuracy are still under researched. Only limited studies have paid attention to the relationship between CF and oral accuracy, showing mixed findings (e.g., Chu, 2011; Rahimpour et al., 2012; Abedi et al., 2015). Thus, one of the three dimensions, accuracy, is the focus of the present study.

Chu (2011) conducted an experimental study about the effects of CF on oral English accuracy in Chinese ESL learners. The study conducted a pre-test and a post-test using class observation and interviewed two CF classes and one control class. The analysis of the recording data showed that the CF classes significantly outperformed the control class, thus proving the substantial positive effect CF had on oral accuracy. Rahimpour et al. (2012) compared the extensive and intensive focus on form strategies (recast and general feedback, respectively) on the oral accuracy of EFL/ESL learners and found no differences between the two types. Abedi et al. (2015) had different findings. Their study found recast was significantly more effective for the oral accuracy of EFL/ESL learners, in comparison with the effects of direct feedback. In short, the efficacy of CF in oral accuracy, a specific dimension of oral production, has still not been extensively examined.

Since the relationship between CF and oral accuracy is still understudied, we tried to explore the effect of learner beliefs of CF on oral accuracy by comparing high-accuracy and low-accuracy learners. In order to compare with EFL/ESL learners’ findings, the current study also addresses five fundamental questions raised by Hendrickson (1978). To sum up, five research questions of the current study are:

In a comparison of CSL learners in a high-accuracy group and CSL learners in a low-accuracy group, are there any differences in their beliefs about (1) the efficacy of CF, (2) the best timing of CF, (3) the types of CF, (4) the types of errors that should be corrected, and (5) the choice of correctors?

## Methodology

### Research Context and Participants

Seventy-six (54 male and 22 female) CSL learners varying in their levels of Chinese participated in the research. They were in a CSL program in a Chinese university. They were selected because they agreed to participate in both the questionnaire survey and the oral test voluntarily. They were following Comprehensive Chinese courses with the aim of developing skills of reading, writing, speaking, and listening by native Chinese teachers. They majored in natural sciences, social sciences and humanities at a university in China, including Accounting, Anthropology, Business, Biology, Chemistry, China Studies, Economics, History, Law, and Music. Their ages ranged from 18 to 35 (mean (M) = 23.6, standard derivation (SD) = 3.46). Their mother tongues included English, French, Italian, German, Dutch, Hebrew, Russian, Spanish, Portuguese, Korean, Japanese, and Indonesian, etc. The average learning time was 22 months, ranging from the longest at 10 years and the shortest at 3 months. Consent from administrators of the institutes was obtained before their participation. All participants provided written informed consent forms and they were assured of the confidentiality and anonymity of the research.

### Data Collection and Analysis

#### Research Instruments

Corrective Feedback Belief Scale (CFBS) (Fukuda, 2004) and a background demographic questionnaire (Zhang and Rahimi, 2014) were translated into Chinese with some adaptations in the current study (Supplementary Appendix). Students were requested to finish those two questionnaires in the lecture within 20 min. CFBS uses a five-point Likert scale, ranging from “strongly agree” (5 points) to “strongly disagree” (1 point), to elicit learners’ beliefs about the provision of CF, the time of providing CF, types of CF, types of errors to correct and the choice of corrections. Cronbach’s α was 0.86, indicating acceptable internal consistency for CFBS (DeVellis, 1991). Unlike Zhang and Rahimi (2014), errors and CF in second language acquisition were not explained systematically before conducting the questionnaire. However, according to their teachers, all students participated in our survey had been provided error corrections in the classroom before. In other words, all of them have perceptual experience of language errors and CF. So their CF beliefs developed naturally with limited intervention, which are the ideal objects we wish to carefully investigate.

#### Oral Test

In order to obtain data on the oral accuracy of each student, an oral test with four topics was conducted for each participant. Participants had 1 min to prepare for each topic before speaking and topics lasted for between 3 and 10 min. Four topics used to elicit oral Chinese were: (1) Please introduce your study and life in Nanjing this semester (up to 3 min); (2) Please introduce your travel experience in China or other places (up to 5 min); (3) Please introduce one of your acquaintances, including his/her appearance, personality, etc. (up to 5 min); and (4) Please compare your hometown and Nanjing, including environment, weather, population, transportation and culture, etc. (up to 10 min). Speech production for those topics was recorded and transcribed into Chinese.

#### Assessment of Oral Accuracy

Each participant’s oral accuracy was assessed in three aspects, i.e., vocabulary, grammar and pronunciation, yielding three independent indicators, i.e., vocabulary accuracy, grammatical accuracy and pronunciation accuracy. Vocabulary accuracy was defined as the ratio of the total number of correctly used words to the total number of words for each participant. Grammatical accuracy was defined as the ratio of the total number of clauses without grammatical errors to the total number of clauses (Jing-Schmidt, 2013). A clause is defined as an independent sentence or a dependent clause of a complex sentence, following the definition of Xing (1997: 13–15) and Jing-Schmidt (2013). In the assessment of vocabulary accuracy and grammatical accuracy, oral data with repetition, self-repair, a false start and pause filler like “en,” “er” were not counted as errors. Based on the above mentioned criteria, all the vocabulary and grammatical errors were labeled exhaustively for each participant by two trained research assistants. While pronunciation accuracy was rated by two experienced Chinese language teachers on a scale of 1 to 10, 1 means “too many errors to understand” while 10 means “native-like pronunciation with very few errors.” The interrater reliability was estimated by Pearson correlation. The ratings were given on an ordinal scale meanwhile the rank orders of the pronunciation performance were essential for us to determine high- and low-accuracy groups. The Pearson correlation coefficient between the two ratings was 0.919 (*p* < 0.001), which means the interrater reliability was strong. The pronunciation accuracy was accordingly established by means of two ratings.

For each indicator of vocabulary accuracy, grammatical accuracy and pronunciation accuracy, we classified the top 25% learners (*n* = 19) as a high-accuracy group and the bottom 25% (*n* = 19) as a low-accuracy group. In total, we obtained 6 groups, namely (I) vocabulary high-accuracy group, (II) vocabulary low-accuracy group, (III) grammatical high-accuracy group, (IV) grammatical low-accuracy group, (V) pronunciation high-accuracy group, and (VI) pronunciation low-accuracy group. The differences between high- and low-accuracy groups with respect to vocabulary (group I vs. group II: *t*(36) = −16.941, *p* < 0.001), grammar (group III vs. group IV: *t*(36) = −13.514, *p* < 0.001) and pronunciation (group V vs. group VI: *t*(36) = −12.993, *p* < 0.001) were all statistically significant.

## Results

To answer five research questions, high- and low-accuracy learners’ responses are reported with regard to their CF beliefs from sections “Research Context and Participants,” “Data Collection and Analysis,” “Research Instruments,” “Oral Test,” and “Assessment of Oral Accuracy.”

### Efficacy of Corrective Feedback

Beliefs on the efficacy of CF were measured by Questions 1 and 2 of the Corrective Feedback Belief Scale (CFBS) in the Supplementary Appendix. With regard to Question 1, 93.4% of 76 participants responded “strongly agree” or “agree” concerning the necessity of error correction. No significant difference was observed between high- and low-accuracy groups in vocabulary (*t*(36) = 1.397, *p* > 0.05), grammar (*t*(36) = 0.577, *p* > 0.05) or pronunciation (*t*(36) = 0.203, *p* > 0.05). This result clearly shows that learners, regardless of their level of oral accuracy, were willing to accept error correction even without any explanation of CF beforehand. This consistency is attributable to learners’ awareness of the benefits of CF on improving their oral accuracy. In other words, participants in the current study were all open to CF. Potential differences between high- and low-accuracy groups were not relevant to their awareness of the necessity of CF.

Question 2 is about the frequency of error correction, and we found that 82.9% of 76 participants preferred their errors to be corrected. Comparing high- and low-accuracy groups, no statistically significant difference was found between high- and low-accuracy groups in vocabulary (*t*(36) = −0.651, *p* > 0.05), grammar (*t*(36) = −0.579, *p* > 0.05) or pronunciation (*t*(36) = 0.262, *p* > 0.05). Learners’ responses to the frequency of CF are in line with their responses to the necessity of CF. It indicates that the level of oral accuracy did not affect learners’ beliefs in the effect of error correction.

### Timing of Corrective Feedback

Questions 4 to 7 elicited learners’ responses to the timing of CF. For 76 participants, “CF after students finish talking” received the highest mean (*M* = 4.17, SD = 0.661), “immediate CF” and “CF after the activity” received the second highest mean (*M* = 3.09, SD = 0.961) and the third highest mean (*M* = 3.05, SD = 0.928), “CF at the conclusion of class” received the lowest mean (*M* = 2.70, SD = 0.994). It indicates that learners generally believe their oral errors should be corrected after they finish talking.

A comparison of high- and low-accuracy groups, learners’ responses are reported in Table 1 with regard to vocabulary, grammar and pronunciation. As shown in the column of “pronunciation” in Table 1, there was no significant difference between high- and low-accuracy groups. Table 1 also illustrates that high- and low-accuracy groups in terms of vocabulary and grammar have significant differences. Low-accuracy groups preferred being corrected after they finished talking more than high-accuracy groups. A possible explanation is that low-accuracy groups normally made more oral errors thus they did not want to be interrupted when talking in order to ensure the entirety of communication.

**TABLE 1:** High- (H) and low-accuracy (L) group responses to the timing of corrective feedback (CF).

Timing of CF	Groups	Vocabulary		Grammar		Pronunciation	
		Mean (SD)	t (*p*)	Mean (SD)	t (*p*)	Mean (SD)	t (*p*)
Immediate CF	L	3.11 (0.809)	0.373 (>0.05)	3.00 (0.745)	0 (>0.05)	3.17 (0.786)	−1.288 (>0.05)
	H	3.00 (0.907)		3.00 (1.155)		3.53 (0.905)	
CF after students finishing talking	L	4.47 (0.612)	2.455 (0.019*)	4.32 (0.582)	2.089 (0.044*)	4.26 (0.562)	1.424 (>0.05)
	H	4.00 (0.577)		3.89 (0.658)		4.00 (0.577)	
CF after the activity	L	3.16 (0.688)	0.183 (>0.05)	3.00 (0.882)	−0.543 (>0.05)	3.16 (0.834)	0.884 (>0.05)
	H	3.11 (1.049)		3.17 (0.985)		2.89 (0.994)	
CF at the conclusion of class	L	2.58 (0.692)	−1.292 (>0.05)	2.16 (0.898)	−3.145 (0.003**)	2.68 (1.057)	−0.396 (>0.05)
	H	2.89 (0.809)		3.00 (0.745)		2.95 (0.621)	

P value less than 0.05 was designated with one asterisk (*).

Moreover, as shown in Table 1, the grammatical high-accuracy group preferred the correction at the conclusion of class more than low-accuracy group. The correction of grammatical errors at the conclusion of class may be beneficial to improve grammatical accuracy because grammatical rules can be generalized at this time.

### Choice of Errors to Correct

Questions 14 to 18 elicit learners’ responses to the types of errors that should be corrected. For all participants, “serious errors” were believed as the most important errors to be corrected (*M* = 4.36, SD = 0.905), followed by “individual errors” (*M* = 4.08, SD = 0.903), “frequent errors” (*M* = 4.07, SD = 0.957) and “less serious errors” (*M* = 3.55, SD = 0.737). “Infrequent errors” were believed as the least important errors to be corrected (*M* = 3.32, SD = 0.927).

Comparing high- and low-accuracy groups, there was no significant difference in vocabulary as shown in Table 2. However, in terms of grammar and pronunciation, mean responses to “infrequent errors” were significantly different between high- and low-accuracy groups. High-accuracy learners in grammar and pronunciation considered “infrequent errors” to be more important than low-accuracy learners.

**TABLE 2:** High- (H) and low-accuracy (L) group responses to types of errors that should be corrected.

Types of errors	Groups	Vocabulary		Grammar		Pronunciation	
		Mean (SD)	t (*p*)	Mean (SD)	t (*p*)	Mean (SD)	t (*p*)
Serious errors	L	4.42 (0.838)	−0.467 (>0.05)	4.21 (0.918)	−0.767 (>0.05)	4.42 (0.769)	0.000 (>0.05)
	H	4.53 (0.513)		4.42 (0.769)		4.42 (0.692)	
Less serious errors	L	3.58 (0.692)	0.248 (>0.05)	3.32 (0.885)	1.816 (>0.05)	3.68 (0.749)	0.226 (>0.05)
	H	3.53 (0.612)		3.79 (0.713)		3.63 (0.684)	
Frequent errors	L	4.21 (0.976)	1.085 (>0.05)	3.89 (1.150)	−0.748 (>0.05)	3.84 (1.015)	−1.417 (>0.05)
	H	3.89 (0.809)		4.16 (1.015)		4.26 (0.806)	
Infrequent errors	L	3.26 (1.046)	−0.997 (>0.05)	3.05 (0.848)	−2.775 (0.009**)	2.95 (0.970)	−2.419 (0.021*)
	H	3.58 (0.902)		3.79 (0.787)		3.63 (0.761)	
Individual errors	L	4.11 (0.875)	−0.019 (>0.05)	3.95 (0.848)	−0.356 (>0.05)	4.06 (0.802)	0.193 (>0.05)
	H	4.11 (0.963)		4.05 (0.970)		4.00 (0.943)	

P value less than 0.05 was designated with one asterisk (*), p value less than 0.01 was designated with two asterisks (**).

### Choice of Corrective Feedback Strategy

As illustrated in Table 3, there were no significant differences in the beliefs of high- and low-accuracy groups about the methods of CF. Learners in all groups rated explicit feedback as the most effective type of CF while recasts were thought to be the least effective type of CF. Fukuda’s (2004) CFBS tested the choice of CF strategies by an error of not using verb past tense in English. When developing the questionnaire in Chinese, since there is no verb conjugation in Chinese, we adapted this verb tense error into a vocabulary error in the Chinese version. However, the vocabulary high- and low-accuracy group did not show any difference in choosing CF strategies toward this vocabulary error.

**TABLE 3:** High- (H) and low-accuracy (L) group responses to corrective feedback (CF) strategies.

CF strategies	Groups	Vocabulary		Grammar		Pronunciation	
		Mean (SD)	t (*p*)	Mean (SD)	t (*p*)	Mean (SD)	t (*p*)
Clarification request	L	3.47 (0.841)	−0.853 (>0.05)	3.47 (0.964)	−1.478 (>0.05)	3.53 (1.020)	−0.174 (>0.05)
	H	3.68 (0.671)		3.84 (0.501)		3.58 (0.838)	
Repetition	L	3.53 (0.697)	0.193 (>0.05)	3.58 (0.838)	0.000 (>0.05)	3.68 (0.820)	1.385 (>0.05)
	H	3.47 (0.964)		3.58 (0.902)		3.32 (0.820)	
Explicit feedback	L	4.32 (0.749)	−0.450 (>0.05)	4.32 (0.885)	0.000 (>0.05)	4.21 (0.787)	−0.444 (>0.05)
	H	4.42 (0.692)		4.32 (0.820)		4.32 (0.671)	
Elicitation	L	3.74 (0.653)	−1.919 (>0.05)	4.05 (0.705)	0.000 (>0.05)	3.95 (0.848)	0.000 (>0.05)
	H	4.21 (0.855)		4.05 (0.621)		3.95 (0.621)	
No corrective feedback	L	4.21 (1.134)	1.144 (>0.05)	4.26 (0.991)	0.367 (>0.05)	4.22 (1.003)	0.035 (>0.05)
	H	3.78 (1.166)		4.16 (0.765)		4.21 (1.032)	
Metalinguistic feedback	L	3.74 (0.872)	−0.396 (>0.05)	3.58 (1.071)	−0.169 (>0.05)	3.47 (1.073)	−0.882 (>0.05)
	H	3.84 (0.765)		3.63 (0.831)		3.74 (0.733)	
Recasts	L	3.11 (1.049)	0.564 (>0.05)	2.84 (1.068)	0.144 (>0.05)	2.79 (1.084)	−0.891 (>0.05)
	H	2.89 (1.243)		2.79 (1.182)		3.11 (1.100)	

### Choice of Correctors

Table 4 illustrates the responses of high- and low-accuracy groups to the choice of correctors. Regardless of vocabulary, grammar or pronunciation, CF from teachers was the most favored, CF by students themselves was the second favored, while their classmates CF was the least favored.

**TABLE 4:** High- (H) and low-accuracy (L) group responses to choice of correctors.

Correctors	Groups	Vocabulary		Grammar		Pronunciation	
		Mean (SD)	t (*p*)	Mean (SD)	t (*p*)	Mean (SD)	t (*p*)
Classmates	L	3.11 (1.049)	0.527 (>0.05)	2.68 (0.946)	−1.474 (>0.05)	3.11 (0.994)	0.808 (>0.05)
	H	2.95 (0.780)		3.11 (0.809)		2.84 (1.015)	
Teachers	L	4.68 (0.478)	0.758 (>0.05)	4.68 (0.478)	−0.722 (>0.05)	4.68 (0.478)	0.000 (>0.05)
	H	4.53 (0.772)		4.79 (0.419)		4.68 (0.478)	
Students themselves	L	4.00 (0.816)	1.102 (>0.05)	3.53 (0.841)	−2.161 (0.037*)	3.84 (0.765)	0.901 (>0.05)
	H	3.68 (0.946)		4.11 (0.809)		3.58 (1.017)	

P value less than 0.05 was designated with one asterisk (*).

Moreover, the grammatical high-accuracy group valued their self-correction significantly higher than the grammatical low-accuracy group. McCormick and Vercellotti (2013) find that grammar is the largest category of self-correction comparing to vocabulary and pronunciation. It seems that grammatical errors are more easily noticed by learners themselves. Grammatical high-accuracy learners, having a lot of overlap with high accuracy learners, should possess more grammar knowledge and are thus more aware of the effectiveness of self-correction of grammar errors, so they value this item more highly than low-accuracy learners.

## Discussion

### Relation Between Learners’ Corrective Feedback Beliefs and Their Second Language Oral Accuracy

Overall, this research shows, regardless of learners’ accuracy level, that there is no significant difference between high- and low-accuracy groups’ CF beliefs in the efficacy and types of CF. It also indicates that vocabulary high- and low-accuracy groups differ in their beliefs about timing of CF; pronunciation high- and low-accuracy groups differ in their beliefs on which type of errors should be corrected; grammatical high- and low-accuracy groups differ in their beliefs regarding timing of CF, which type of errors should be corrected and the choice of correctors. The discrepancy between high- and low-accuracy groups’ CF beliefs suggests that these beliefs are related to speakers’ oral accuracy. High-accuracy speakers have some unique CF beliefs such as the preference of correction at the conclusion of class and the preference of correction of infrequent errors. Previous research has found that learner beliefs can directly affect their learning behavior, and further influence their learning outcomes (Mori, 1999; Borg, 2003). Our results confirm that learners’ CF beliefs can modulate their language accuracy. From the perspective of language pedagogy, those results can have potential implications for improving learners’ accuracy. Specifically, the discrepancy between high- and low-accuracy groups’ CF beliefs further suggests that in providing CF, learners’ accuracy levels should be taken into account by the teacher. This confirms Ellis (2009b) guidelines for CF that “teachers should be prepared to vary who, when and how they correct in accordance with cognitive and affective needs of the individual learner.” For example, teachers should pay more attention to frequent errors for low-accuracy learners while infrequent errors for high-accuracy learners. It is also suggested that teachers can leave correction until the end of fluency practice for high-accuracy leaners as they also expect to develop their fluency and pragmatic competence (Gong et al., 2021a).

Additionally, the results of this study suggest that three indicators of oral accuracy, i.e., vocabulary, grammar and pronunciation, have different relations to CF beliefs. Students have different learning strategies for vocabulary, grammar and pronunciation and thus develop different CF beliefs for those aspects. For example, the acquisition of vocabulary is less interconnected and internalized than the acquisition of grammar and pronunciation. Learners can improve their accuracy of grammar and pronunciation by correcting infrequent errors. This may explain why high-accuracy groups of grammar and pronunciation considered infrequent errors more important than their low-accuracy peers, respectively. But such a difference was not found between high- and low-accuracy groups in vocabulary. This result suggests that teachers should implement a variety of CF strategies when teaching vocabulary, grammar and pronunciation in oral communication and integrate opportunities and resources outside the classroom to improve students’ communicative competence (Gong et al., 2021c). Corrections of infrequent errors in grammar and pronunciation are potentially valuable to high-accuracy learners.

### Comparison With Corrective Feedback Beliefs of EFL/ESL Learners

Our results firstly showed that CSL learners of the current study shared many CF beliefs with EFL/ESL learners. For example, participants of the current study also showed strong support for the frequent provision of CF, even without a prior explanation of the purpose and significance of CF. They generally believed serious errors and frequent errors should be corrected. They also ranked explicit feedback as the best method of CF and ranked the teacher as the favorite choice of correctors. Learners of different languages and from different regions have developed those common CF beliefs probably because second language learners have realized the efficacy of those CF strategies during the process of language acquisition.

However, CSL participants of the current study differ from EFL/ESL participants in their beliefs about the timing and provider of CF. For the best timing of CF, our participants chose “CF after students finish talking,” i.e., delay correction, in comparison to “as soon as errors are made.” First, questions used in our questionnaire resolve the ambiguity of “delay correction” and “immediate correction” as noted by Zhu and Wang (2019) by clearly noting when CF is given (see section “Timing of Corrective Feedback”). We further compare our results with Zhang and Rahimi (2014) because we use the same questionnaire as they did. While Zhang and Rahimi (2014) find the best timing of CF is “immediate CF,” our participants preferred “CF after students finish talking”. What causes the difference in beliefs about timing of CF between the two studies? One justification for this difference is that participants of the two studies were taking different courses and thus had different expectations for the timing of oral correction. Learners in oral communication classes probably preferred immediate correction while learners in other classes preferred correction after they finished talking. Zhang and Rahimi (2014) distributed the questionnaire in an oral communication course. In the oral course, students focused on improving their speaking skills so they expected their oral errors to be corrected without any delay. However, our survey was conducted in comprehensive Chinese courses. Students not only practiced their speaking skills but also their reading, listening and writing skills in the class. Thus they wanted to express their meaning completely without interruption. Teachers need to take into account the course type when deciding whether to correct immediately or not, because learners’ expectation of immediate error correction is probably higher in an oral communication course than in other types of courses.

Moreover, our study found peer correction was the least favored and self-correction was the second favored, while in Schulz (2001), Zhang and Rahimi (2014), Agudo (2015), and Zhu and Wang (2019), participants preferred peer correction more than self-correction. This difference can be attributed to the accessibility of peer correction. Participants of Schulz (2001) were Colombian students; participants of Zhang and Rahimi (2014) were Persian EFL learners studying in Iran; participants of Agudo (2015) were Spanish EFL secondary school students; participants of Zhu and Wang (2019) were Chinese university students. What they have in common is that participants were from the same countries and shared similar language backgrounds. On the one hand, students from the same countries may know more about the language problems in their peers’ oral production and know how to correct these errors. On the other hand, it can be speculated that participants in the above four studies should have plenty of social connection after class. However, participants of the current study originated from 28 different countries and spoke 23 different mother tongues. They are speculated to have less contact outside the classroom, or more social isolation (Sawir et al., 2008; Gong et al., 2021b), than participants of Schulz (2001), Zhang and Rahimi (2014), Agudo (2015), and Zhu and Wang (2019). Having classmates with different language backgrounds and cultural backgrounds may reduce students’ expectations of the CF from classmates. As a result, participants of the current study relied more on self-correction than peer correction. The implication for CSL teaching is that CSL learners in China may depend more on self-correction than peer-correction if there is less contact with their classmates or language partners after class. In this case, the teacher should suggest appropriate and adequate references about target language to facilitate learners’ self-correction.

Generally speaking, our results show that CSL and EFL/ESL learners share many common CF beliefs. The differences between them have little to do with the target language of learning but are more relevant to the research design. Therefore, many language pedagogies developed from the research of EFL/ESL learners’ CF beliefs such as Ellis (2009b) should be able to shed light on this area and have significance for CSL learners.

## Conclusion

Our research questions sought to explore the differences in high-accuracy and low-accuracy learners’ CF beliefs from a cohort of CSL learners. The research attempts to examine the relationship between CSL learners’ CF beliefs and oral accuracy, by adopting a questionnaire survey and an oral test with 76 CSL learners from a Chinese university. The results highlight that high- and low-accuracy learners of CSL share many CF beliefs like the efficacy of CF and CF strategies, but also differ in timing of CF, error types and choice of correctors. Learners also show different CF beliefs in terms of vocabulary, grammar and pronunciation. Those results provide direct implications for correcting learner errors in teaching CSL.

In addition, the current study also investigates if and why CF beliefs of CSL and EFL/ESL learners differ. Our findings suggest that common CF beliefs are the mainstream while minor differences in the timing and provider of CF exist. We attribute those differences to research design rather than different mechanism in learning Chinese and English. Further investigations should control CSL and EFL/ESL learners’ course type and language background in order to confirm our speculations.

It must be noted that our investigation was only conducted with CSL learners in China, and any generalization of the findings to all CSL learners worldwide should be undertaken with caution. This study was based on self-reported questionnaire. It would be helpful to carry out interviews to understand the reasons behind learner’s CF beliefs in future research. There are also several potential topics to be explored in future research. For example, another possible study could investigate how the unique CF beliefs held by the high-accuracy group affect their achievement of high accuracy in language performance. Additionally, a future study might recruit more participants to its sample to examine whether participants’ origin countries and mother tongues influence their CF beliefs, although they studied Chinese in the same environment. Lastly, future research could also investigate, besides accuracy, whether the other two core dimensions of second language acquisition, i.e., fluency and complexity, interact with CF beliefs.

## Data Availability Statement

The raw data supporting the conclusions of this article will be made available by the authors, without undue reservation.

## Ethics Statement

The studies involving human participants were reviewed and approved by the Institute for International Students, Nanjing University. The patients/participants provided their written informed consent to participate in this study.

## Author Contributions

JZ: conceptualization, methodology, formal analysis, resources, writing and funding acquisition. XC: conceptualization, methodology, resources, and funding acquisition. NZ: methodology, resources, and investigation. All authors approved the submitted version.

## Conflict of Interest

The authors declare that the research was conducted in the absence of any commercial or financial relationships that could be construed as a potential conflict of interest. The handling Editor (YFG) declared a shared affiliation with the author (JZ) at the time of review.

## Publisher’s Note

All claims expressed in this article are solely those of the authors and do not necessarily represent those of their affiliated organizations, or those of the publisher, the editors and the reviewers. Any product that may be evaluated in this article, or claim that may be made by its manufacturer, is not guaranteed or endorsed by the publisher.

## References

[B1] Abedi M. Karimi L. Mehrdad A. G. (2015). Comparing the effects of recast vs. direct feedback on EFL students’ pronunciation accuracy. *Int. J. Educ. Investig.* 2 159–169.

[B2] Agudo J. (2015). How do Spanish EFL learners perceive grammar instruction and corrective feedback? *Southern African Linguist. Appl. Lang. Stud.* 33 1–15. 10.2989/16073614.2015.1061890 33685377

[B3] Bartram M. Walt R. (1991). *Correction. Mistake Management: A Positive Approach for Language Teachers.* Hove: Language Teaching Publications.

[B4] Benson S. DeKeyser R. (2019). Effects of written corrective feedback and language aptitude on verb tense accuracy. *Lang. Teach. Res.* 23 702–726. 10.1177/1362168818770921

[B5] Bitchener J. Young S. Cameron D. (2005). The effect of different types of corrective feedback on ESL student writing. *J. Second Lang. Writ.* 14 191–205. 10.1016/j.jslw.2005.08.001

[B6] Borg M. (2003). Teacher cognition language teaching: a review of research on what teachers think, know, believe, and do. *Lang. Teach.* 36 81–109. 10.1017/S026144480300190

[B7] Chong S. W. (2019). A systematic review of written corrective feedback research in ESL/EFL contexts. *Lang. Educ. Assess.* 2 70–95. 10.29140/lea.v2n2.138

[B8] Chu R. (2011). Effects of teacher’s corrective feedback on accuracy in the oral English of English-majors college students. *Theory Pract. Lang. Stud.* 1 454–459. 10.4304/tpls.1.5.454-459

[B9] Davis A. (2003). Teachers’ and students’ beliefs regarding aspects of language learning. *Eval. Res. Educ.* 17 207–222. 10.1080/09500790308668303

[B10] DeVellis R. F. (1991). *Scale Development: Theory and Application.* Thousand Oaks, CA: Sage Publications.

[B11] Doughty C. Varela E. (1998). “Communicative focus on form,” in *Focus on Form in Classroom Second Language Acquisition*, eds Doughty C. Williams J. (New York, NY: Cambridge University Press), 114–138.

[B12] Ellis R. (2009a). A typology of written corrective feedback types. *English Lang. Teach. J.* 63 97–107. 10.1093/elt/ccn023

[B13] Ellis R. (2009b). Corrective feedback and teacher development. *L2 J.* 1 3–18. 10.5070/l2.v1i1.9054

[B14] Ellis R. (2009c). The differential effects of three types of task planning on the fluency, complexity, and accuracy in L2 oral production. *Appl. Linguist.* 30 474–509. 10.1093/applin/amp042

[B15] Ellis R. (2010). A framework for investigating oral and written corrective feedback. *Stud. Second Lang. Acquis.* 32 335–349. 10.1017/S0272263109990544

[B16] Ellis R. Loewen S. Erlam R. (2006). Implicit and explicit corrective feedback and the acquisition of L2 grammar. *Stud. Second Lang. Acquis.* 28 339–368. 10.1017/S0272263106060141

[B17] Ellis R. Sheen Y. Murakami M. Takashima H. (2008). The effects of focused and unfocused written corrective feedback in an English as a foreign language context. *System* 36 353–371. 10.1016/j.system.2008.02.001

[B18] Ferris D. (1999). The case for grammar correction in l2 writing classes: a response to Truscott (1996). *J. Second Lang. Writ.* 8 1–10. 10.1016/S1060-3743(99)80110-6

[B19] Frear D. Chiu Y. H. (2015). The effect of focused and unfocused indirect written corrective feedback on EFL learners’ accuracy in new pieces of writing. *System* 53 24–34. 10.1016/j.system.2015.06.006

[B20] Fukuda Y. (2004). *Treatment of Spoken Errors in Japanese High School Oral Communication Classes*, Master’s thesis. San Francisco, CA: State University.

[B21] Gong Y. Gao X. Lyu B. (2020a). Teaching Chinese as a second or foreign language to non-Chinese learners in mainland China (2014-2018). *Lang. Teach.* 53 44–62. 10.1017/S0261444819000387

[B22] Gong Y. Guo Q. M. Lai C. Wang C. (2021a). Developing literacy or focusing on interaction: New Zealand students’ strategic efforts related to Chinese language learning during study abroad in China. *System* 98:102462. 10.1016/j.system.2021.102462

[B23] Gong Y. Gao X. Li M. Lai C. (2021b). Cultural adaptation challenges and strategies during study abroad: New Zealand students in China. *Lang. Cult. Curriculum* 34 417–437. 10.1080/07908318.2020.1856129

[B24] Gong Y. Lai C. Gao X. (2021c). Language teachers’ identity in teaching intercultural communicative competence. *Lang. Cult. Curriculum* 1–17. 10.1080/07908318.2021.1954938

[B25] Gong Y. Lai C. Gao X. (2020b). The teaching and learning of Chinese as a second or foreign language: the current situation and future directions. *Front. Educ. China* 15:1–13. 10.1007/s11516-020-0001-0

[B26] Han Y. (2017). Mediating and being mediated: learner beliefs and learner engagement with written corrective feedback. *System* 69 133–142. 10.1016/j.system.2017.07.003

[B27] Han Y. Hyland F. (2015). Exploring learner engagement with written corrective feedback in a Chinese tertiary EFL classroom. *J. Second Lang. Writ.* 30 31–44. 10.1016/j.jslw.2015.08.002

[B28] Han Z.-H. (2002). A study of the impact of recasts on tense consistency in L2 output. *TESOL Q.* 36 543–572. 10.2307/3588240

[B29] Harmer J. (1983). *The Practice of English Language Teaching.* London: Longman.

[B30] Hashemifardnia A. Namaziandost E. Sepehri M. (2019). The effectiveness of giving grade, corrective feedback, and corrective feedback-plus-giving grade on grammatical accuracy. *Int. J. Res. Stud. Lang. Learn.* 8 15–27. 10.5861/ijrsll.2019.3012

[B31] Hendrickson J. (1978). Error correction in foreign language teaching: recent theory. *Res. Practice Modern Lang. J.* 62 387–398. 10.2307/326176

[B32] Jing-Schmidt Z. (2013). From interlanguage development research to CFL curriculum design: implications of research on content-based instruction. *Chin. Teach. World* 27 144–155. 10.13724/j.cnki.ctiw.2013.01.010

[B33] Kalaja P. Barcelos A. M. F. Aro M. (2018). “Revisiting research on l2 learner beliefs: looking back and looking forward,” in *The Routledge Handbook of Language Awareness*, eds Garrett P. Cots J. M. (Oxfordshire: Routledge Handbooks in Linguistics), 222–237.

[B34] Karim K. Nassaji H. (2020). The revision and transfer effects of direct and indirect comprehensive corrective feedback on ESL students’ writing. *Lang. Teach. Res.* 24 519–539. 10.1177/1362168818802469

[B35] Katayama A. (2007). Students’ perceptions of oral error correction. *Japanese Lang. Literature* 41 61–92. 10.2307/30198022

[B36] Kern R. G. (1995). Students’ and teachers’ beliefs about language learning. *Foreign Lang. Ann.* 28 71–92. 10.1111/j.1944-9720.1995.tb00770.x

[B37] Kim Y. Emeliyanova L. (2021). The effects of written corrective feedback on the accuracy of L2 writing: comparing collaborative and individual revision behavior. *Lang. Teach. Res.* 25 234–255. 10.1177/1362168819831406

[B38] Krashen S. (1982). *Principles and Practice in Second Language Acquisition.* Oxford: Pergamon.

[B39] Krashen S. (1985). *The Input Hypothesis: Issues and Implications.* London: Longman.

[B40] Lee E. (2013). Corrective Feedback Preferences and Learner Repair among Advanced ESL Students. *System* 41 217–230. 10.1016/j.system.2013.01.022

[B41] Leki I. (1991). The preferences of ESL students for error correction in college level writing classes. *Foreign Lang. Ann.* 24 203–218. 10.1111/j.1944-9720.1991.tb00464.x

[B42] Li S. (2010). The effectiveness of corrective feedback in SLA: a meta-analysis. *Lang. Learn.* 60 309–365. 10.1111/j.1467-9922.2010.00561.x

[B43] Li S. Zhu Y. Ellis R. (2016). The Effects of the timing of corrective feedback on the acquisition of a new linguistic structure. *Modern Lang. J.* 100 276–295. 10.1111/modl.12315

[B44] Loewen S. Li S. Fei F. Thompson A. Nakatsukasa K. Ahn S. (2009). Second language learners’ beliefs about grammar instruction and error correction. *Modern Lang. J.* 93 91–104. 10.1111/j.1540-4781.2009.00830.x

[B45] Long M. (1996). “The role of the linguistic environment in second language acquisition,” in *Handbook of Second Language Acquisition*, eds Ritchie W. Bhatia T. (San Diego, CA: Academic Press), 413–468. 10.1016/b978-012589042-7/50015-3

[B46] Long M. (2006). “Recasts in SLA: the story so far,” in *Problems in SLA*, ed. Long M. H. (Mahwah, NJ: Laurence Erlbaum), 75–116.

[B47] Lyster R. Ranta L. (1997). Corrective feedback and learner uptake: negotiation of form in communicative classrooms. *Stud. Second Lang. Acquis.* 19 37–66. 10.1017/S0272263197001034

[B48] Lyster R. Saito K. (2010). Oral feedback in classroom SLA: a meta-analysis. *Stud. Second Lang. Acquis.* 32 265–302. 10.1017/S0272263109990520

[B49] Mackey A. Goo J. (2007). “Interaction research in SLA: a meta-analysis and research synthesis,” in *Conversational Interaction in Second Language Acquisition: A Series of Empirical Studies*, ed. Mackey A. (Oxford: Oxford University Press), 407–453. 10.1371/journal.pone.0053509

[B50] McCormick D. E. Vercellotti M. L. (2013). Examining the impact of self-correction notes on grammatical accuracy in speaking. *TESOL Q.* 47 410–420. 10.1002/tesq.92

[B51] Mori Y. (1999). Epistemological beliefs and language learning beliefs: what do language learners believe about their learning? *Lang. Learn.* 49 377–415. 10.1111/0023-8333.0009

[B52] Oladejo J. (1993). Error correction in ESL: learners’ preferences. *TESL Canada J.* 10 71–89. 10.18806/tesl.v10i2.619

[B53] Panova I. Lyster R. (2002). Patterns of corrective feedback and uptake in an adult ESL classroom. *TESOL Q.* 36 573–595. 10.2307/3588241

[B54] Powell L. A. (1987). *The Effects of Learner Control versus Program Control of Corrective Feedback on Listening Comprehension and Vocabulary Assimilation of Low versus High Performers in Beginning College Spanish*, [Doctoral dissertation]. Columbus: The Ohio State University.

[B55] Quinn P. (2014). *Delayed Versus Immediate Corrective Feedback on Orally Produced Passive Errors in English*, [Unpublished Ph.D. dissertation]. Toronto: University of Toronto.

[B56] Rahimpour M. Salimi A. Farrokhi F. (2012). The impact of extensive and intensive focus on form strategies on EFL learners’ oral accuracy. *Int. J. Appl. Linguist. English Literature* 1 37–43. 10.7575/ijalel.v.1n.6p.37

[B57] Sawir E. Marginson S. Deumert A. Nyland C. Ramia G. (2008). Loneliness and international students: an Australian study. *J. Stud. Int. Educ.* 12 148–180. 10.1177/1028315307299699

[B58] Schulz R. A. (2001). Cultural differences in student and teacher perceptions concerning the role of grammar instruction and corrective feedback. *Modern Lang. J.* 85 244–258. 10.1111/0026-7902.00107

[B59] Sheen Y. (2007). The effect of focused written corrective feedback and language aptitude on ESL learners’ acquisition of articles. *TESOL Q.* 41 255–283. 10.1002/j.1545-7249.2007.tb00059.x

[B60] Sheen Y. (2011). *Corrective Feedback, Individual Differences and Second Language Learning.* New York, NY: Springer.

[B61] Sheen Y. Ellis R. (2011). “Corrective feedback in the language teaching,” in *Handbook of Research in Second Language Teaching and Learning*, ed. Hinkel E. (New York, NY: Routledge), 593–610.

[B62] Shintani N. Aubrey S. (2016). The effectiveness of synchronous and asynchronous written corrective feedback on grammatical accuracy in a computer-mediated environment. *Modern Lang. J.* 100 296–319. 10.1111/modl.12317

[B63] Skehan P. (1989). *Individual Differences in Second-language Learning.* London: Edward Arnold.

[B64] Skehan P. (1996). A framework for the implementation of task-based instruction. *Appl. Linguist.* 17 38–62. 10.1093/applin/17.1.38

[B65] Skehan P. (1998). *A Cognitive Approach to Language Learning.* England: Oxford University Press.

[B66] Storch N. Wigglesworth G. (2010). Learners’ processing, uptake, and retention of corrective feedback on writing: case studies. *Stud. Second Lang. Acquis.* 32 303–334. 10.1017/S0272263109990532

[B67] Truscott J. (1996). The case against grammar correction in l2 writing classes. *Lang. Learn.* 46 327–369. 10.1111/j.1467-1770.1996.tb01238.x

[B68] Truscott J. (1999). The case for “the case for grammar correction in l2 writing classes”: a response to Ferris. *J. Second Lang. Writ.* 8 111–122. 10.1016/S1060-3743(99)80124-6

[B69] VanPatten B. (1992). Second-language acquisition research and foreign language teaching, part 2. *ADFL Bull.* 23 23–27. 10.1632/adfl.23.3.23

[B70] Walker J. L. (1973). Opinions of university students about language teaching. *Foreign Lang. Ann.* 7 102–105. 10.1111/j.1944-9720.1974.tb00088.x

[B71] Xing F. (1997). *Chinese Grammar.* Changchun: Northeast Normal University Press.

[B72] Xinhua News Agency (2020). *The Accumulative Total Number of Chinese Learners outside China Reached 200 Million and Online Education Tools Like “Chinese Plus” Were Released.* Available online at: http://world.people.com.cn/n1/2020/0906/c1002-31851012.html [accessed November 20, 2021]

[B73] Yoshida R. (2008). Learners’ perception of corrective feedback in pair work. *Foreign Lang. Ann.* 41, 525–541. 10.1111/j.1944-9720.2008.tb03310.x

[B74] Zhang L. J. Rahimi M. (2014). EFL learners’ anxiety level and their beliefs about corrective feedback in oral communication classes. *System* 42 429–439. 10.1016/j.system.2014.01.012

[B75] Zhu Y. Wang B. (2019). Investigating english language learners’ beliefs about oral corrective feedback at chinese universities: a large-scale survey. *Lang. Awareness* 28 139–161. 10.1080/09658416.2019.1620755

