# Supplementary material for: How Learners’ Corrective Feedback Beliefs Modulate Their Oral Accuracy: A Comparative Study on High- and Low-Accuracy Learners of Chinese as a Second Language
Source: Front Psychol. 2022 May 11;13:869468. doi: 10.3389/fpsyg.2022.869468 (PMC9132164; doi:10.3389/fpsyg.2022.869468)
Supplement: Supplementary file 1 [file Data_Sheet_1.pdf]

Appendix

纠错反馈信念量表

Corrective Feedback Belief Scale

说明：

请回想您对于汉语学习的个人感受。仔细阅读每一道题目，选出最符合您的感受的选项。选项没有正确和错误之分，只是是否符合您的情况。您可以跳过不想作答的题目。

Instructions:

Please reflect on your personal feelings regarding Chinese learning. Carefully read each statement and indicate to what extent you agree or disagree by circling the statement that best describes how you feel. There are no right or wrong answers, just those that are accurate to your situation. You may skip any question you feel uncomfortable answering.

- 请圈出最符合你的一个选项。

Please circle the information that applies to you. Please mark only one.

1. 当我犯错时，我想得到纠错反馈。（例如：给我提供一个线索来进行自我纠正，提醒我犯了错误，或者纠正我的错误）

非常同意　同意　一般　不同意　强烈反对

I want to receive corrective feedback when I make mistakes. (e.g., provide a hint for me to self-correct, tell me that I made an error, or correct my error)

Strongly agree　Agree　Neutral　Disagree　Strongly disagree

2. 你希望老师对你的口语错误多长时间进行一次纠错反馈？

总是　经常　有时候　偶尔　不要

How often do you want your teacher to give corrective feedback on your spoken errors?

Always (100%)　Usually (80%)　Sometimes (50%)　Occasionally (20%)　Never (0%)

- 你希望你的口语错误什么时候被纠正?

At which moment would you want your spoken errors to be corrected?

3. 刚一犯错误就被纠正，即使会打断我说话。

非常有效　有效　一般　没有效果　非常没有效果

As soon as errors are made, even if it interrupts my speaking.

Very Effective　Effective　Neutral　Ineffective　Very Inefficient

4. 在我说完之后。

非常有效　有效　一般　没有效果　非常没有效果

After I finish speaking.

Very Effective　Effective　Neutral　Ineffective　Very Inefficient

5. 在整个口语练习结束之后。

非常有效　有效　一般　没有效果　非常没有效果

After the entire speaking exercise is finished.

Very Effective　Effective　Neutral　Ineffective　Very Inefficient

6. 在课堂总结的时候。

非常有效 有效 一般 没有效果 非常没有效果

At the conclusion of class.

Very Effective Effective Neutral Ineffective Very Inefficient

- 请阅读下面这段对话：

Please read the following conversation:

老师：你多大的时候开始学习汉语？

学生：我十八年开始学习汉语。

在这段对话中，学生犯了口语错误。你怎样评价下面的口语纠错方法？

The student spoke incorrectly. How would you rate each of the following methods of correcting spoken errors?

7. 你可以再说一遍吗？

非常有效 有效 一般 没有效果 非常没有效果

Could you say that again?

Very Effective Effective Neutral Ineffective Very Inefficient

8. 十八年？（重复：老师通过改变他/她的声调来强调学生的语法错误。）

非常有效 有效 一般 没有效果 非常没有效果

十八年? (Repetition: The teacher emphasizes the student's grammatical error by changing his/her tone of voice.)

Very Effective Effective Neutral Ineffective Very Inefficient

9. "年"是用来表示时间的。这里你要说的是年龄大小，应该用"岁"。（直接反馈：老师告诉学生正确的形式，并进行语法解释。）

非常有效 有效 一般 没有效果 非常没有效果

"年" is used to describe time. You need to use "岁" here to express age. (Explicit feedback: The teacher gives the correct form to the student with a grammatical explanation.)

Very Effective Effective Neutral Ineffective Very Inefficient

10. 我十八……（启发式：老师让学生来纠正和完成这个句子。）

非常有效 有效 一般 没有效果 非常没有效果

我十八……(Elicitation: The teacher asks the student to correct and complete the sentence.)

Very Effective Effective Neutral Ineffective Very Inefficient

11. 真的吗？那你已经学了好几年了。（没有纠错反馈：老师不对学生的错误进行纠错反馈。）

非常有效 有效 一般 没有效果 非常没有效果

Really? You have studied Chinese for several years. (No corrective feedback: The teacher does not give corrective feedback on the student's error.)

Very Effective Effective Neutral Ineffective Very Inefficient

12. 当我们说年龄大小的时候，要用什么？（元语言反馈：老师给出提示和线索，不特别指出错误。）

非常有效 有效 一般 没有效果 非常没有效果

What measure word do we use when talk about age? (Metalinguistic feedback: The teacher gives a hint or a clue without specially pointing out the mistake.)
Very Effective Effective Neutral Ineffective Very Inefficient
13. 我十八岁开始学习汉语。(重铸：老师用正确的形式重复学生的表达，不指出学生的错误。)
非常有效 有效 一般 没有效果 非常没有效果
我十八岁开始学习汉语。(Recast: The teacher repeats the student's utterance in the correct form without pointing out the student's error.)
Very Effective Effective Neutral Ineffective Very Inefficient

- 你希望以下类型的口语错误多长时间得到一次纠错反馈？

How often do you want each of the following types of spoken error to receive corrective feedback?
14. 严重的口语错误，会影响别人理解的。
总是 经常 有时候 偶尔 不要
Serious spoken errors that may cause problems in a listener's understanding.
Always (100%) Usually (80%) Sometimes (50%) Occasionally (20%) Never (0%)
15. 不太严重的口语错误，不会影响别人理解的。
总是 经常 有时候 偶尔 不要
Less serious spoken errors that do not affect a listener's understanding.
Always (100%) Usually (80%) Sometimes (50%) Occasionally (20%) Never (0%)
16. 经常出现的口语错误。
总是 经常 有时候 偶尔 不要
Frequent spoken errors.
Always (100%) Usually (80%) Sometimes (50%) Occasionally (20%) Never (0%)
17. 不经常犯的口语错误。
总是 经常 有时候 偶尔 不要
Infrequent spoken errors.
Always (100%) Usually (80%) Sometimes (50%) Occasionally (20%) Never (0%)
18. 我个人的错误。(例如：别的学生不会犯的错误)
总是 经常 有时候 偶尔 不要
My individual errors (i.e., errors that other students may not make.)
Always (100%) Usually (80%) Sometimes (50%) Occasionally (20%) Never (0%)

- 你认为下面的哪些人可以纠正学生的口语错误？

Which people are most appropriate to correct the student?
19. 同学
非常同意 同意 一般 不同意 强烈反对
Classmates
Strongly agree Agree Neutral Disagree Strongly disagree
20. 老师
非常同意 同意 一般 不同意 强烈反对

Teachers
Strongly agree Agree Neutral Disagree Strongly disagree

21. 自己

非常同意 同意 一般 不同意 强烈反对
Myself
Strongly agree Agree Neutral Disagree Strongly disagree
